# Current efforts in regional climate planning: A dataset from Italian NUTS2 regions

**DOI:** 10.1016/j.dib.2024.111223

**Published:** 2024-12-17

**Authors:** Luigi Santopietro, Filomena Pietrapertosa, Angela Pilogallo, Monica Salvia

**Affiliations:** a Institute of Methodologies for Environmental Analysis – National Research Council of Italy, C.da S. Loja, 85050 Tito Scalo, PZ, Italy; b NBFC, National Biodiversity Future Center, Palermo 90133, Italy

**Keywords:** Mitigation, Adaptation, Climate-change, Ambition, Robustness, Implementation and progress, Transnational initiative

## Abstract

This data article provides a comprehensive description of climate change mitigation and adaptation policies implemented by 21 Italian regions (NUTS2 level) as of January 2024. It was developed as part a wider research work published by the authors [2].

The dataset collects information on the efforts the regions are making to tackle the climate crisis. In particular, it contains a collection of regional climate plans (RCPs) and a catalogue of their contents analysed with regard to objectives, planned actions and monitoring and evaluation indicators.

To complete the regional framework, the dataset also provides an overview of the socio-economic data for the Italian regions, derived from EUROSTAT (as of 2023), and the climate indicators from the Italian CIRO (Climate Indicators for Italian Regions) database, updated to 2021. Regional sustainable development strategies were also examined for consistency with climate planning and climate emergency declarations.

Moreover, specific data are presented on the regions' participation in transnational networks and initiatives, as well as references on climate legislation currently in force (January 2024).

Specifications Table

**Table:** 

Subject	Planning and Development, Renewable Energy, Sustainability and the Environment, Environmental Engineering.
Specific subject area	Analyses and evaluation with a systemic approach of the efforts made by Italian NUTS2 regions in the field of climate change adaptation and mitigation policies.
Type of data	.xlsx file (data tables and metadata) Filtered
Data collection	Data retrieved for RCPs were searched on the web (both regional and national institutional portals) using the following keywords (in Italian): [name of the region] + climate + plan; [name of the region] + mitigation + plan; [name of the region] + adaptation + plan; [name of the region] + adaptation + strategy. The content analysis of climate plans was carried out by means of guiding questions mainly based on [1]. Participation in transnational networks and regional climate laws were searched for on institutional websites.
Data source location	Italian NUTS2 regions
Data accessibility	Repository name: Supplementary material for “Current efforts in regional climate planning: a dataset from Italian NUTS2 regions Data identification number: 10.17632/nz37swhhzv.1 Direct URL to data: https://data.mendeley.com/datasets/nz37swhhzv/1
Related research article	Monica Salvia, Angela Pilogallo, Luigi Santopietro, Filomena Pietrapertosa, A methodological framework for assessing regional climate efforts. Learning from its application in Italy, Journal of Cleaner Production, 2024, 144,299, ISSN 0959–6526, 10.1016/j.jclepro.2024.144299. (https://www.sciencedirect.com/science/article/pii/S095965262403748X).

## 1 Value of the Data

• Focus on regional climate planning: Data offers valuable insights in the field of regional climate planning. Specifically, they provide an overview of the bridging role pursuit by the regional authorities, between the municipalities and the States, in facing climate change processes and playing a pivotal function in the governance processes.
• Flexible methodological approach: Data collection is the result of a flexible methodological approach replicable in other countries but also on different scales, taking into account the availability of data. Broader analysis can be performed on data gathered with various approaches in describing the efforts of regions climate planning.
• Overview of mitigation and adaptation regional commitments: The data provide a detailed picture of the climate change mitigation and adaptation commitments undertaken at the Italian NUTS2 level. An overview of the various commitments made (updated to January 2024) provides a baseline for monitoring the actions undertaken.
• Replicability and extension of the dataset: Researchers can utilize the data as baseline to provide a regional perspective of the mitigation and adaptation measures. These data are the first step of a non-exhaustive dataset, which can be extended through additional information and results by forthcoming studies.

## 2 Background

This dataset analyses and evaluates with a systemic approach the efforts made by Italian regions in the field of climate change adaptation and mitigation policies [2]. The reasons for the interest of this multi-case study are manifold. Firstly, Italy lies in the centre of the Mediterranean basin, widely recognized as one of the most vulnerable areas to climate change [3]; secondly, 53 % of Italian municipalities (4261) are located in inner areas are particularly exposed to risk [4,5].

A multiple-case study approach [6] was adopted to investigate the efforts made by the Italian regions in mitigating and adapting to climate change. Firstly, a monitoring overview of the various commitments undertaken so far (updated to January 2024) in climate action was drawn up.

Although the study offers a country-specific view, the proposed methodological framework and content analysis methods can be replicated to obtain a comprehensive picture of the current efforts of local authorities (e.g. municipalities, provinces, regions) in any other country in climate mitigation and adaptation planning.

## 3 Data Description

RCP data are collected in a workbook with a total of 7 sheets. 6 sheets collect data and information from the RCPs and other regional data investigated, while one sheet describes the metadata provided.

The “Regional Climate Policies” sheet is divided into 6 sections:

—Adaptation and mitigation sections

**Fig. 1:**
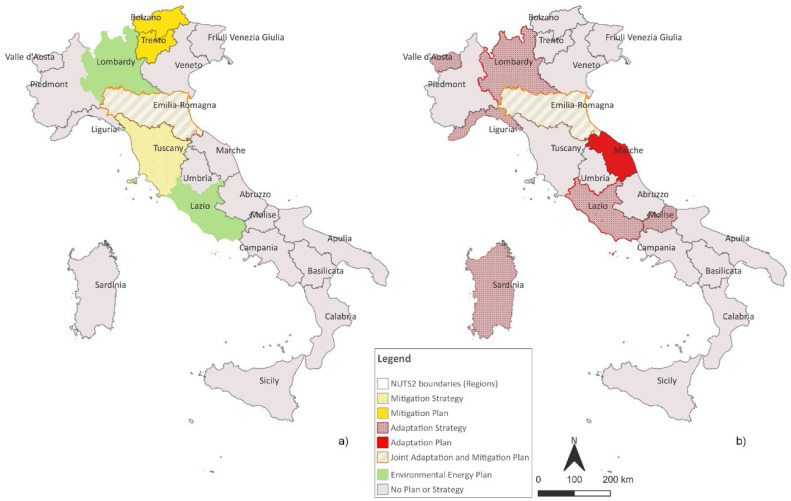
On the left Italian regions with a mitigation regional plan, on the right with an adaptation one.

The first two sections provide an overview of the mitigation/adaptation plans for the Italian regions classified according to EUROSTAT NUTS2 codes. For each region the name of the plan (in the national language and in English), the approval year, the website and the monitoring plan (if available) are given (Fig. 1.). It was also checked whether the plan had been established as part of an EU project, whether it had benefited from support from external scientific institutions and consultancies, and whether it had included a participatory process in its development. For each plan the link to the regional sustainable development strategy and possible joint approach between adaptation and mitigation plan were investigated.

—GHG Emissions Inventory

**Fig. 2:**
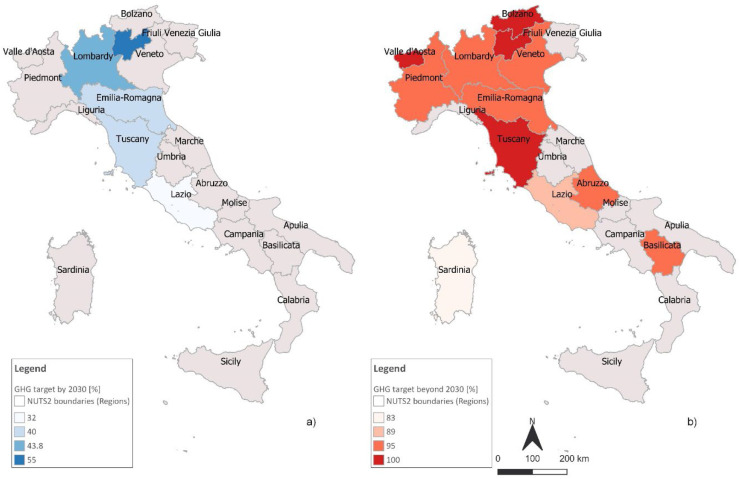
GHG target set in the mitigation RCPs by 2030 (a) and beyond 2030 (b).

This section focuses on the GHG emission reduction targets, target year and baseline emission year set in the mitigation RCPs (Fig. 2.) and/or in light of the commitments made within the Under2 MoU Coalition, which are to “pursue emission reductions consistent with a trajectory of 80–95 % below 1990 levels by 2050 and/or achieve a per capita annual emissions target of less than 2 tonnes by 2050”.

—Regional Sustainable Development Strategy (RSDS)

The RSDS aims to identify the main instruments to contribute to the achievement of the goals of the National Strategy for Sustainable Development (NSSD) as well as to the goals and targets contained in the 2030 Agenda for Sustainable Development Resolution adopted in 2015 by the United Nations General Assembly.

**Fig. 3:**
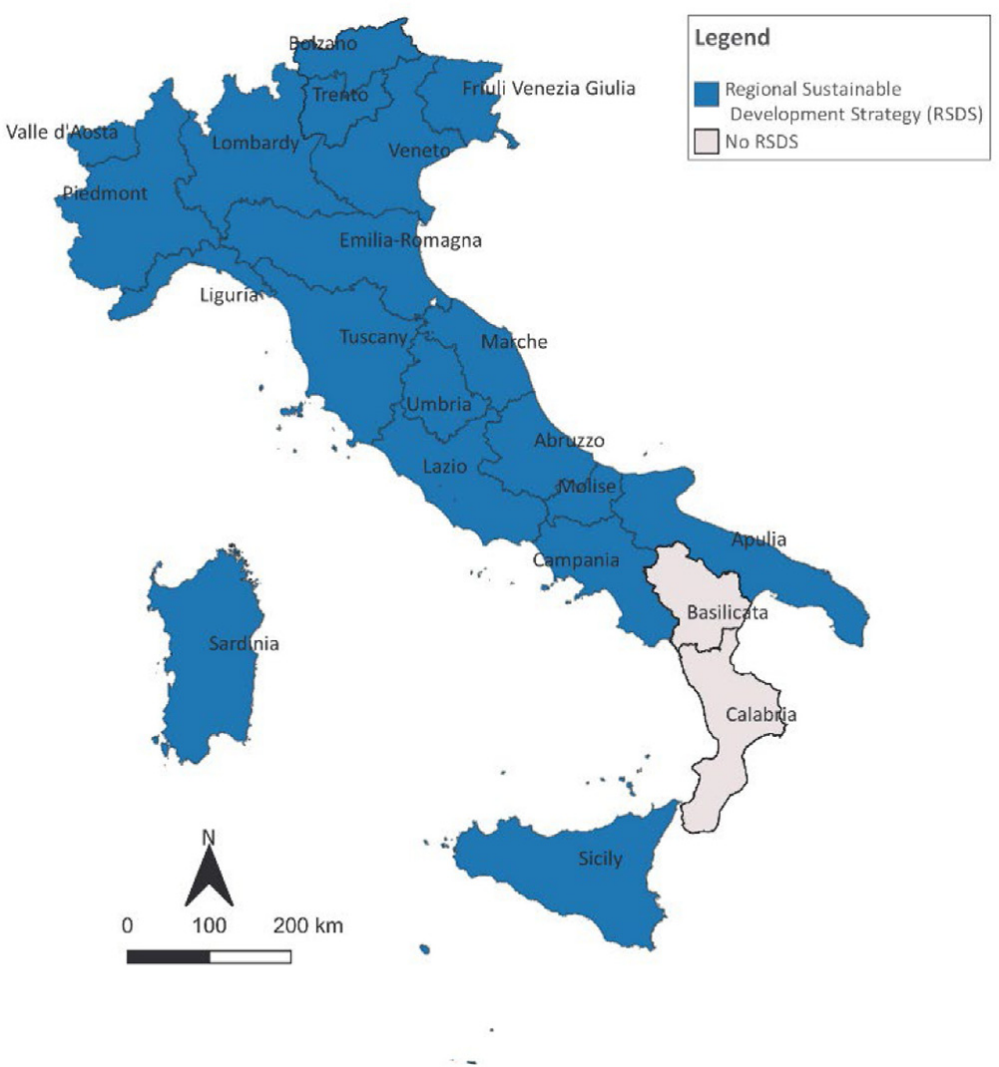
Overview of the Italian regions with a regional sustainable development strategy.

The RSDS approved by each region (Fig. 3) was collected on the basis of national sources (or by the institutional portals) and then cross-referenced and updated as of January 2024 through ad hoc web searches in each regional institutional portal.

—Transnational regional networks and initiatives

**Fig. 4:**
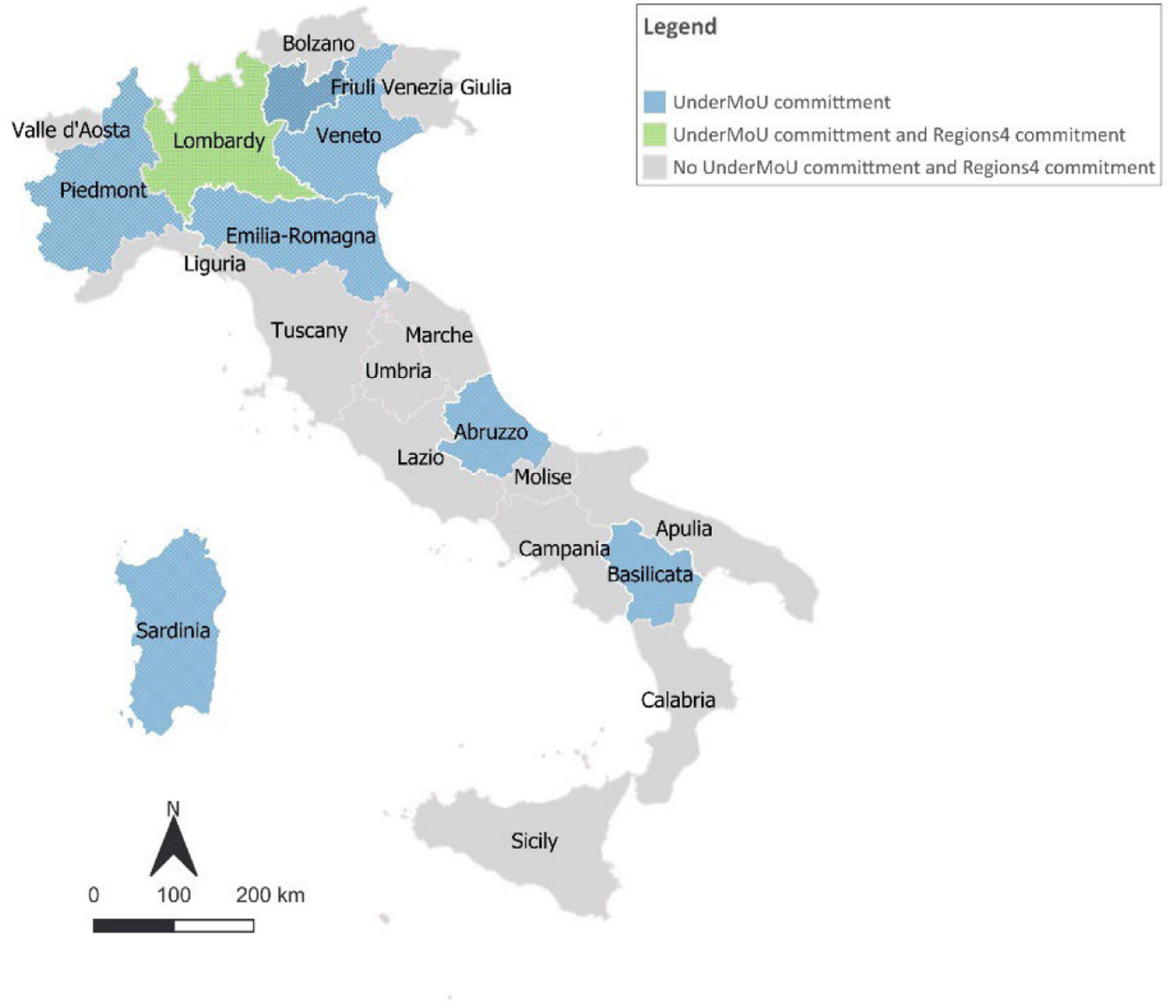
Under2MoU and Regions4 commitments set by Italian regions.

**Fig. 5:**
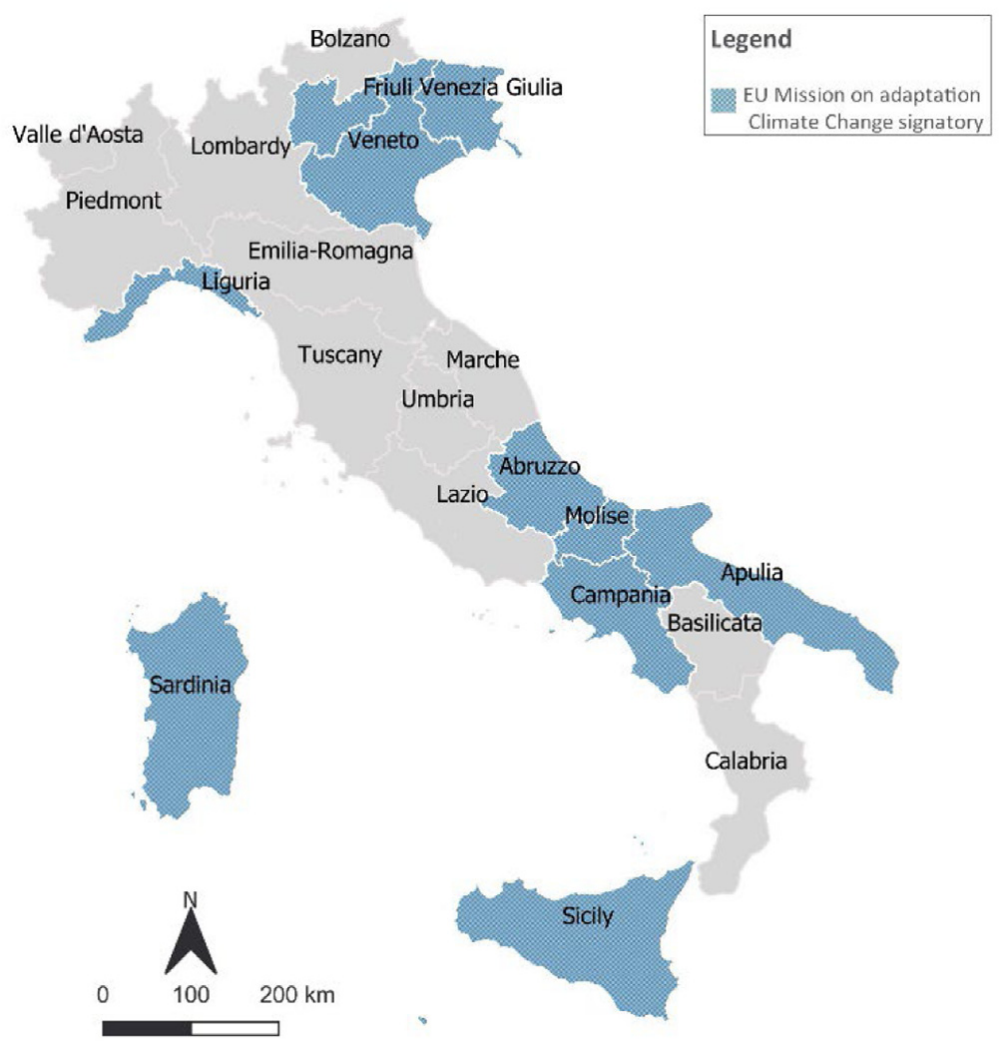
Mission on adaptation Climate Change signatories.

The dataset includes information on the partnership of Italian regions within the major transnational regional networks and initiatives (Fig. 4, Fig. 5). In particular, the Under2 Coalition, a global network of subnational climate actors, who, by signing the Under2 Memorandum of Understanding (MOU)^1^ express their voluntary commitment to reduce greenhouse gas emissions by 80–95 % by 2050. This coalition is linked to other three initiatives and networks: Race to Zero, Climate Ambition Alliance, and Race to Resilience. Regions4, a network of regional governments to enable them to create and strengthen connections and translate them into impactful actions, in particular on adaptation (RegionsAdapt).

The dataset also includes information on the participation to the recent EU Mission on Adaptation to Climate Change, which supports regional and local authorities in their efforts to build resilience against the impacts of climate change by taking into account different climate vulnerabilities and levels of preparedness.

—Climate Emergency Declaration

**Fig. 6:**
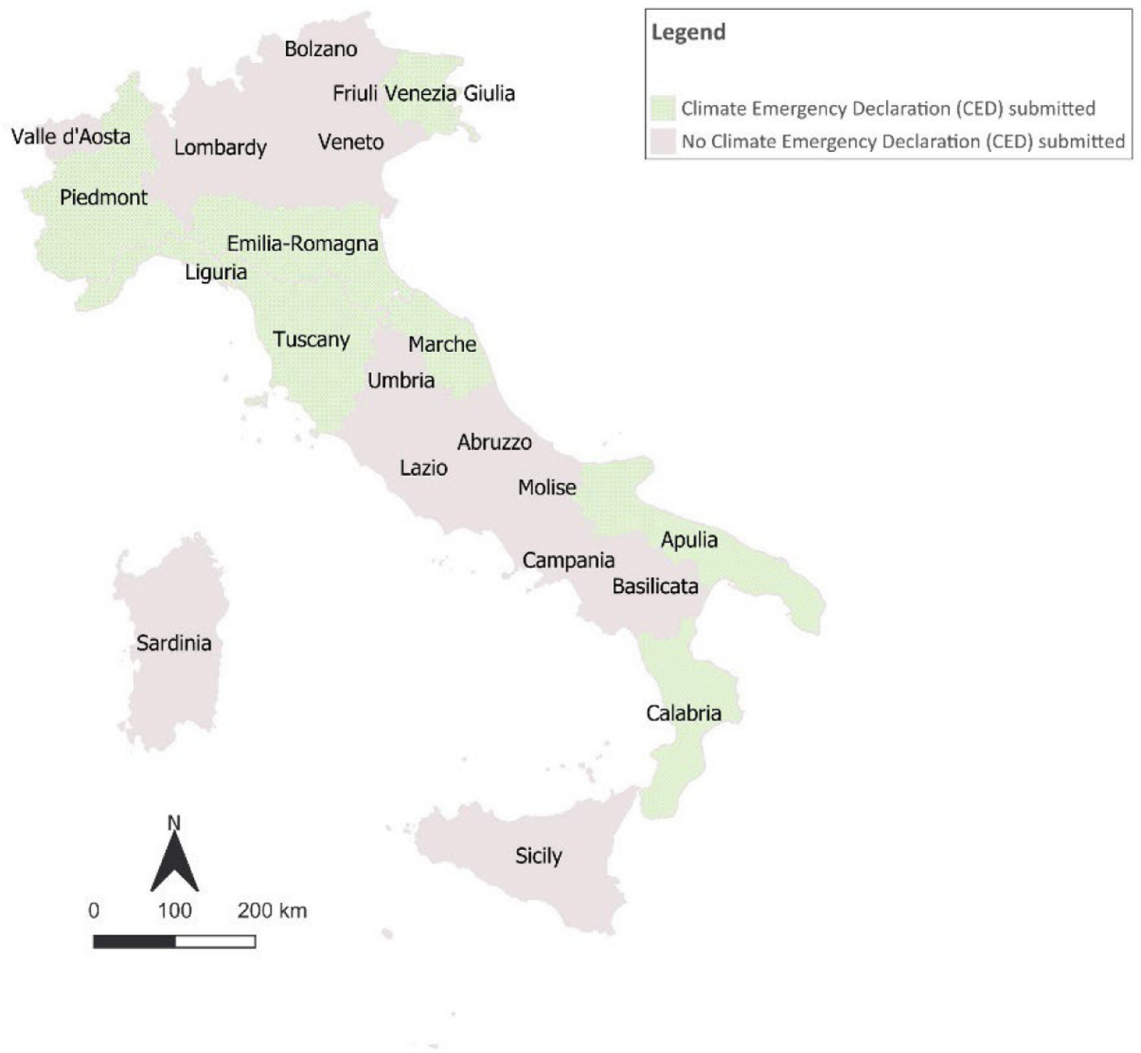
Italian regions with a Climate Emergency Declaration (CED) submitted.

The endorsement of Climate Emergency Declarations (CED) by regional (or provincial) authorities (Fig. 6), which seems to help motivate municipalities to become more ambitious in climate action was considered a pre-commitment mainly for mitigation [7]. The main source for CEDs is the Climate Emergency Declaration and Mobilisation in Action - CEDAMIA dataset, which witnesses the ʻbottom-upʼ demand for more action on climate change.

—NAZCA DB

This section takes into account the participation of each region to the UN Framework Convention on Climate Change (UNFCCC) platform that brings together commitments and actions of the Non-State Actor Zone for Climate Action (NAZCA). The NAZCA platform of the UNFCCC was launched in December 2014. NAZCA showcases the vast range of actions pledged and underway by non-state actors (e.g. businesses, investors and civil society organisations) and sub-national actors (e.g. cities and regions). These actions range from emission reduction pledges and renewable energy production commitments to internal carbon pricing and green bond investments.

“Additional Dataset”

It includes a heterogeneous set of climate, social and energy data provided at NUTS2 level. It is divided in 2 sections:

—EUROSTAT data includes:

i. The heating degree day (HDD), a weather-based technical index designed to describe the need for the heating energy requirements of buildings. HDD data are presented as C temperature sums.
ii. The cooling degree day (CDD), a weather-based technical index designed to describe the need for the cooling (air-conditioning) requirements of buildings. CDD data are presented as C temperature sums.
iii. Gross domestic product (GDP) at current market prices by EUROSTAT NUTS 2 regions.
iv. Number of residents of each NUTS2 region expressed in relation to its area.

Bearing in mind that climate change will affect energy demand for heating and cooling of buildings, it is interesting to characterise the Italian regions in terms of heating degree days (HDD) and cooling degree days (CDD). Again, a strong variation was observed between northern, southern and island regions. Heating degree days, in fact, vary between 3960 HDD in the Autonomous Province of Bolzano and 971 HDD in Sardinia (national average: 1734 HDD). The situation is reversed, however, when looking at cooling degree days, where values range from 572 CDD in Sardinia to about 6 in Valle d'Aosta (national average: 375 CDD).

—CIRO (Climate Indicators for Italian RegiOns) an online database, realised by the company "Italy for Climate"
in collaboration with ISPRA, includes:

i. Per capita greenhouse gas emissions.
ii. Final energy consumption per capita.
iii. Share of energy consumption from renewables.

“Analysis of adaptation content” sheet

This datasheet provides information on the content of the adaptation strategies and/or plans adopted by the Italian regions collected by searching into the plans for the answers to the questions reported in the dataset. It reports whether the adaptation plans identify a concrete adaptation objective with respect to a specific impact/risk, and quantitative adaptation objectives established in the sector categories of the National Adaptation Plan (NAP). This sheet also reports whether plans envisaged measures in the following 13 sectors (Urban settlements; Transport; Energy; Water; Waste; Agriculture and forestry; Environment, greenery, biodiversity; Health; Social and educational institutions and services; Tourism; Industries; Marine ecosystems and coastal area; cultural heritage); whether plans refer to the development or use of disaster response systems based on civil protection network, whether plans mention vulnerable population groups, whether plans include at least one measure to support the approval of SECAPs; whether plans include measures on strengthening knowledge about CC and its impacts; whether include measures aimed at mainstreaming CC adaptation within planning instruments at the several scales and whether time-bound actions for quantitative adaptation goals are included.

“Monitoring and Evaluation” sheet

This datasheet reports data on the types of indicators used in the plans, classified according to the subdivision made in the NAP, Progress and/or Effectiveness; or that foreseen by the NAP for the regional level (Process, Contribution and Context). In addition, this tab contains data on the presence of M&E indicators in the 18 NAP sectors (Transports; Energy; Water resources; Agriculture; Forests; Sea fishing; Aquaculture; Geological, hydrological and hydraulic instability; Inland and transitional water ecosystems; Marine Ecosystems; Terrestrial Ecosystems; Urban Settlements; Cultural Heritage; Health; Tourism; Desertification; Coastal areas; Hazardous; Infrastructure and Industries).

“RVA & NAP Sectors” sheet

With regard to adaptation, dataset investigated the availability of a Risk and Vulnerability Assessment (RVA) study for each region, either within or separate from the adaptation RCP to assess how climate-related hazards and their interactions may affect regional territories. Where an RVA was available, the content analysis investigated the method chosen to develop the RVA and the vulnerability areas addressed, according to the Italian NAP classification

“Regional Climate Laws”

The pathway of Italian climate policies varies greatly between regions. In some cases, the approved plan (both for mitigation and adaptation) is the end point of the regional legislative framework. In other cases it represents an intermediate step and the regional governments have further legislated to broaden climate objectives including adaptation or pursuing a more integrated approach with multiple dimensions of environmental sustainability and management (e.g. pollution, circular economy and waste management).

On the mitigation side, 6 Regions declared a mitigation target. Among these, however, 4 Regions (Tuscany, Lombardy, Emilia Romagna, Bolzano) approved a proper Mitigation Plan while Lazio and Trento (AP) defined their target within a regional energy plan.

Some other regions, such as Basilicata, Calabria, Campania and Umbria, have only recently (respectively in 2018, 2022 2024 and 2018) undertaken the path of climate policies, approving regional laws that provide guidelines for drafting climate plans. These pathways are still in progress and have not yet been finalised.

On the adaptation side, 8 regions approved a mitigation plan between 2016 (Lombardy) and 2023 (Liguria and Marche). There are two main paths of climate policies for adaptation. The first is that of the regions Liguria, Lombardy and Sardinia, which have independently approved their own adaptation plan. The second, instead, characterises Lazio, Marche, Molise and Valle d'Aosta, which have drawn up their own adaptation plan as part of the wider roadmap envisaged for the Regional Strategy for Sustainable Development.

## 4 Experimental Design, Materials and Methods

The experimental design of the study is related to the efforts made by Italian regions in the field of climate change adaptation and mitigation policies. The dataset included has been structured on data deriving from mitigation and adaptation plans provided by the 21 regions on their institutional websites. Data on the GHG emission reduction targets, target year and baseline emission year set in the mitigation RCPs and/or within the Under2 MoU Coalition.

A comprehensive overview on sustainable development commitment at regional level was provided by the collection of the RSDS approved by each region on the basis of national source [8] and then cross-referenced and updated as of January 2024 through ad hoc web searches in each regional institutional portal.

Participation in transnational regional networks and initiatives was retrieved from the websites of the network analysed (Under2Mou, Regions4 and the EU Mission On Adaptation to Climate Change). In order to collect regional CEDs, Climate Emergency Declaration and Mobilisation in Action, the CEDAMIA dataset was examined. The engagement of regions in the Non-State Actor Zone for Climate Action (NAZCA) was assessed through the verification of their inclusion in the UNFCCC platform.

Further information on climate, social, economic and energy - environment characteristics were also added to the “Additional dataset” datasheet.

The indicators used to define the climatic characteristics of the regions are the Heating Degree Day (HDD) index a weather-based technical index designed to describe the need for the heating energy requirements of buildings and the Cooling degree days (CDD) index, a weather-based technical index designed to describe the severity of the heat in a specific time period taking into consideration outdoor temperature and average room temperature. They are retrieved from EUROSTAT [9] and presented on NUTS-2 level.

GDP and population density retrieved from EUROSTAT [9] are used to describe the economic and social characteristics at NUTS-2 level.

Energy-environment characteristics include the greenhouse gas emissions produced and final energy consumption per inhabitant and share of energy consumption from renewables. They are extracted from the CIRO (Climate Indicators for Italian RegiOns) database, realised by Italy for Climate in collaboration with Italian Institute for Environmental Protection and Research (ISPRA) [10].

The availability of a regional adaptation strategy and/or plan was reported using Boolean values. The adaptation content analysis was carried out by searching for indicators within the collected strategies/plans. The indicators were designed as guiding questions and are mainly based on [1]. Results are reported in relation to NAP sectors.

The “monitoring and evaluation” datasheet describes the availability of monitoring indicators in the regional adaptation plans collected through the content analysis. The indicators identified in the plans have been classified according to the NAP classification, in terms of Progress and/or Effectiveness; and according to that provided by the NAP for the regional level (Process, Contribution and Context) expressed by Boolean values. The same datasheet collects data on the M&E indicators identified in the regional adaptation plans in each of the 18 NAP sectors, using Boolean values.

“RVAs and NAPs” datasheet lists the existing risk and vulnerability assessment (RVA) studies for the Italian regions. The RVAs were collected through content analysis of the plans and through region-specific web searches. They are presented using Boolean values.

“Regional climate law” datasheet collected a comprehensive overview of the current status of regional climate legislation. The collection was carried out through web searches (on both regional and national institutional portals).

## Limitations

One of the main limitations concerns the geographical coverage of the dataset, as it relates to a specific case study that provides an overview of the actions that the 21 Italian regions are taking to address climate change issues.

Another limitation of the data collection is that it focuses only on climate policies identified as such and does not provide data on climate measures and policies that may have been included in specific sectoral plans.

Likewise, as data on the climate change legislation was searched using keywords such as “climate change mitigation” and “climate change adaptation”, the information collected could be not exhaustive. As climate change is a cross-cutting issue that affects all of a region's development policies, climate policies may have been also included in additional legislation that was not consulted.

Furthermore, it is important to emphasise that the data collected is based on a set of indicators that the authors selected as the more suitable choice to analyse certain aspects of their research, but many other aspects could be analysed by defining other specific indicators.

Finally, the content analysis was carried out only on the mitigation and adaptation plans, not on the regional sustainable development strategies or climate change legislation.

## Ethics Statement

All authors have read and follow the ethical requirements for publication in Data in Brief and confirming that the current work does not involve human subjects, animal experiments, or any data collected from social media platforms.

## CRediT Author Statement

**Luigi Santopietro:** Data collection and curation, Investigation and Visualization, Writing - original draft, Writing – review & editing. **Filomena Pietrapertosa:** Supervision, Conceptualization, Writing - original draft, Writing – review & editing, Data collection and curation. **Angela Pilogallo:** Data collection and curation, Conceptualization, Investigation and Visualization, Writing - original draft, Writing – review & editing, Data collection and curation. **Monica Salvia:** Supervision, Conceptualization, Data collection and curation, Investigation and Visualization, Writing - original draft, Writing – review & editing.

## Data Availability

Mendeley DataSupplementary material for “Current efforts in regional climate planning: a dataset from Italian NUTS2 regions” (Original data).

## Footnotes

1 More information is available at website https://www.theclimategroup.org/under2-memorandum-understanding.
